# Cerebrospinal fluid and plasma β‐endorphin levels in children with cerebral malaria

**DOI:** 10.1002/brb3.673

**Published:** 2017-03-17

**Authors:** Oluwatosin Eunice Olorunmoteni, Oluwagbemiga Oyewole Adeodu, Saheed B. A. Oseni, Efere M. Obuotor

**Affiliations:** ^1^ Department of Paediatrics and Child Health Obafemi Awolowo UniversityIle‐IfeOsun StateNigeria; ^2^ Department of BiochemistryObafemi Awolowo UniversityIle‐IfeOsun StateNigeria

**Keywords:** beta‐endorphin, cerebral malaria, cerebrospinal fluid, plasma

## Abstract

**Objectives:**

Cerebral malaria (CM) is the most lethal form of malaria, yet its pathogenesis is not fully understood. Cytoadherence, sequestration, alterations in cytokine expression, inflammation, and microvascular obstruction are all hypothesized to be important in the aetio‐pathogenesis of coma which characterizes cerebral malaria and the death which sometimes result. Beta (β)‐endorphin has been postulated to be involved in the pathogenetic processes of inflammation and cytokine expression, although the exact role is unknown. The aim of this study was to determine the levels of β‐endorphin in cerebrospinal fluid (CSF) and plasma of children with CM and compare the levels of β‐endorphin in the plasma of children with CM with that of apparently healthy age‐ and sex‐matched controls at Ile‐Ife, Nigeria.

**Materials and Methods:**

Additional to the standard investigation for CM, CSF and venous blood samples were obtained from the subjects for the determination of β‐endorphin levels.

**Results:**

Forty children with CM were studied along with forty age‐ and sex‐matched controls. The mean CSF β‐endorphin (± SD) level for the children with CM was 1.8 ± 0.9 pmol/L. The mean plasma β‐endorphin levels at admission (3.1 ± 2.0 pmol/L) and discharge (4.1 ± 3.3 pmol/L) were higher in children with CM than in the control subjects (2.7 ± 0.7 pmol/L). However, only the mean plasma β‐endorphin levels at discharge was significantly higher than that of controls (*p *=* *.012).

**Conclusion:**

Children with CM had higher mean plasma β‐endorphin levels compared to the controls and there was increased production of β‐endorphins in children with CM during the course of the illness.

## 1 Introduction

Malaria is a protozoan disease that is known to be prevalent in many countries of the world. It is endemic in the tropics and subtropics with 96 countries having ongoing malaria transmission in 2015 (World Health Organization 2015). According to the World Health Organization (WHO), there were an estimated 214 million cases of malaria in 2015 with about 88% of these cases occurring in Africa (African Heads of State and Government, 2000; World Health Organization 2015). About 90% of all the malaria deaths in 2015 occurred in the WHO African region (African Heads of State and Government, 2000; World Health Organization, 2015). In Nigeria, malaria accounts for about 60% of outpatient visits, 30% of hospital admissions and about 25% of under‐five mortality (Ogun, 2006; Orimadegun, Fawole, Okereke, Akinbami, & Sodehinde, 2007).

CM is an acute febrile, rapidly progressive, diffuse encephalopathy in which *Plasmodium falciparum* parasitemia is accompanied by altered consciousness in the absence of other coma etiologies (Division of Control of Tropical Diseases, 1990). It is the most lethal form of severe malaria with most cases occurring in children below 5 years (Division of Control of Tropical Diseases, 1990). It contributes about 20%–30% of pediatric severe malaria cases with case fatality rates ranging between 5% and 50% (Division of Control of Tropical Diseases, 1990; Phillips & Solomon, 1990). However, the role of β‐endorphins in its pathogenesis and outcome has not been fully elucidated.

Beta‐endorphin (β‐endorphin) is an endogenous opioid peptide synthesized in the brain which has been found to play a role in analgesia and neuroendocrine regulation (Frederickson & Geary, 1982). It also functions in autonomic regulation, modulation of cardiovascular responses and cerebral blood flow (Frederickson & Geary, 1982; Koneru, Satyanarayana, & Rizwan, 2009; Molina, 2006; Stoupel, Pinchas, Gilad, Laron, & Agmon, 1986). Factors that trigger β‐endorphin release include stress, hypoxia, acidosis (Koneru et al., 2009; Molina, 2006; O’çonnor, O'halloran, & Shanahan, 2000) and inflammation (Blalock, 2005; Brack et al., 2004; Machelska & Stein, 2000; O’çonnor et al., 2000). Since these trigger factors for increased β‐endorphin levels are associated with the pathophysiology of cerebral malaria, we hypothesized that β‐endorphin level may be raised in CSF and plasma in CM. There is no report in the literature on β‐endorphin levels in CM. However, β‐endorphin level has been reported to be elevated in some CNS conditions such as aseptic meningitis (Nagamitsu, 1993), status epilepticus (Calabrese et al., 1993) and the Rett syndrome (Echenne, Bressot, Bassir, Daures, & Rabinowitz, 1991). It has also been reported that there is a negative correlation between the CSF β‐endorphin levels in the above neurologic disorders and increasing age (Nagamitsu, 1993). With previously highlighted direct relationship between β‐endorphin levels and the above CNS diseases, it could be assumed that β‐endorphin may have a role to play in the pathogenesis of cerebral malaria as a consequence of the inflammatory response and cytokine expression seen in CM.

We determined the levels of β‐endorphin in CSF and plasma of children with CM and compared the plasma levels of β‐endorphin in the plasma of children with that of apparently healthy age‐ and sex‐matched controls. We chose to use apparently healthy age‐ and sex‐matched children as controls because these children are not exposed to the trigger factors for β‐endorphin production such as acidosis, fever and hypoxia which are likely to be present in the subjects with CM. It was thought that the result from the study will aid further understanding of the role; if any, of β‐endorphin in the pathogenesis and outcome of CM.

## 2 Materials and Methods

### 2.1 Case selection

There were two groups of participants recruited into this comparative study. In the first group (subjects) were 40 children with a diagnosis of cerebral malaria aged between six months and fourteen years and fulfilled the WHO diagnostic criteria for cerebral malaria as follows (Division of Control of Tropical Diseases, 1990): (1) unarousable coma (Blantyre Coma Score ≤2) for more than 30 min; (2) asexual forms of *P. falciparum* parasitemia; (3) exclusion of other common causes of loss of consciousness (such as meningitis, head trauma).

The lower age limit of the study population represents the age below which malaria is uncommon while the upper limit is the cutoff age for pediatric care according to the hospital policy. Children who had history suggestive of underlying chronic medical conditions such as chronic kidney disease, sickle cell anemia, cerebral palsy, seizure disorder etc. were excluded as well as those whose parents or guardians did not give consent to participate in the study.

In the second group (controls) were 40 children who were recruited from the children outpatient clinic and were matched individually for age and sex with the children with CM. They were apparently well children who were in clinic for preschool commencement medical examination or medical checkups. Only those whose parents or guardians did not give consent were excluded.

Ethical approval for the study was granted by the OAUTHC Ethics and Research Committee. Informed consent was obtained using information and consent form. Recruitment of subjects and controls was done consecutively until the sample size was reached.

### 2.2 Data collection

A research proforma designed for the study was used to obtain relevant information from the subjects. The level of consciousness was determined using the Blantyre Coma Scale which is applicable to children including preverbal ones (Molyneux, Taylor, Wirima, & Harper, 1989). Children with CM having a score of two or below (out of the maximum score of five) were recruited for the study while the controls were conscious and healthy children. Detailed neurologic examination was done including checking for signs of meningeal irritation, corneal reflex, pupillary size and reflex, indirect ophthalmoscopy, cranial nerve function, muscle power, muscle tone, deep tendon reflexes, and posture. Indirect ophthalmoscopy was done by one of the authors (O.E.) having had special training on ophthalmoscopy for a month at the Ophthalmology Unit of the hospital. It was done using a handheld Gowllands Coeydon (UK) ophthalmoscope after dilating the pupils with 1% Tropicamide (Tropic acid‐N‐ethyl‐N‐(γ‐picolyl)‐amide). Funduscopic findings were categorized as normal, retinal whitening, vessel whitening, retinal hemorrhage, and papilledema.

### 2.3 Investigation

Blood was taken from the subjects at admission for the following investigations: blood film for malaria parasite, β‐endorphin measurement, random blood glucose, serum electrolytes, urea, creatinine and full blood count and bedside hematocrit. Lumbar puncture was done on subjects with CM as a routine procedure to exclude meningitis. Blood was taken from each of the subjects with CM at admission and discharge for the measurement of β‐endorphin level. The controls were not admitted. One milliliter of blood was taken from controls for the measurement of their β‐endorphin levels at recruitment. These blood samples were collected in ethylene‐diamine tetra‐acetic acid (EDTA) bottles and centrifuged at 1000g for 15 min. The plasma was separated and stored together with the CSF sample at −80°C before analysis.

CSF and plasma β‐endorphin levels were determined using ELISA kit E90806Hu obtained from USCN Life Sciences, China at a research laboratory located at the Department of Biochemistry, Obafemi Awolowo University (OAU) Ile‐Ife. The other CSF and blood samples were analyzed in the microbiology, hematology, and chemical pathology laboratories of the OAUTHC as appropriate.

### 2.4 Treatment

Intravenous artesunate was given for six days or until the patient regained consciousness and thereafter changed to a complete course of oral artemisinin‐based combination therapy if the patient could tolerate orally (World Health Organization, 2010). Other supportive treatments were given as necessary. At discharge, another detailed neurologic assessment was done by the investigator to identify neurologic sequelae. The outcome of illness was classified into three categories as follows: full recovery, recovery with neurologic sequelae, and death.

### 2.5 Data analysis

Data were analyzed using the Statistical Program for Social Sciences (SPSS) software for Windows version 16.0.

## 3 Results

### 3.1 Demographic characteristics and clinical features

The age range (in completed months) of the study subjects and controls was between eight and 84 months with a mean age of 32.9 ± 18.6 months. There were 25 (62.5%) males and 15 (37.5%) females among both the subjects with CM and controls giving a male to female ratio of 1.7: 1.

### 3.2 Clinical symptom and signs at admission

Apart from coma and fever which was present in all the subjects, the other common clinical features include: convulsions (85.0%), pallor (45.0%), and deep acidotic breathing (35.0%).

#### 3.2.1 Neurologic findings

Eleven (27.5%) children had Blantyre coma score (BCS) of one or less while three (7.5%) had profound coma (BCS of 0). Absent cornea reflex was observed in 22 (55.0%) of the subjects. Malaria retinopathy was observed in about one‐third of children with CM (35.0%). These findings included: papilledema (2.5%), retinal hemorrhage (12.5%), retinal whitening (7.5%), and vessel whitening (12.5%).

### 3.3 Laboratory results

#### 3.3.1 Plasma β‐endorphin levels in subjects and controls

##### Plasma β‐endorphin levels in the subjects and controls

The mean (± SD) plasma β‐endorphin level at admission for the subjects was 3.1 ± 2.0 pmol/L with a range of 1.0–10.4 pmol/L while the mean (± SD) plasma β‐endorphin level at discharge was 4.1 ± 3.3 pmol/L with a range of 1.1–14.4 pmol/L. Five out of the 40 subjects with cerebral malaria died during the course of treatment. Therefore, blood sample at discharge was only taken from 35 subjects.

The mean (± SD) plasma β‐endorphin level for the controls (at recruitment) was 2.7 ± 0.7 pmol/L with a range of 1.2–3.7 pmol/L.

##### Plasma β‐endorphin levels of subjects with CM (at admission and discharge) and controls

Table 1 shows a lack of statistically significant difference between the mean plasma β‐endorphin level of the subjects at admission and the controls (*p *=* *.209). However, a statistically significant difference was found between the mean plasma β‐endorphin level of the subjects with CM at discharge and the controls (*p *=* *.012).

**Table 1:** Plasma β‐endorphin levels of the subjects with CM (at admission and discharge) and the controls

Variables	Mean plasma β‐endorphin levels		*t* value	*p* value
	Subjects with CM	Controls		
At admission				
Males	2.6 ± 1.7	2.7 ± 0.7	−0.11	.917
Females	3.9 ± 2.4	2.7 ± 0.7	1.91	.067
Total population	3.1 ± 2.0	2.7 ± 0.7	1.27	.209
At discharge				
Males	3.4 ± 2.7	2.7 ± 0.7	1.41	.165
Females	5.2 ± 4.2	2.7 ± 0.7	2.35	.027
Total population	4.1 ± 3.3	2.7 ± 0.7	2.59	.012

##### Differences in mean plasma β‐endorphin levels at admission and discharge among the cerebral malaria group

The mean plasma β‐endorphin level was higher at discharge (4.1 ± 3.3 pmol/L) than at admission (3.1 ± 2.0 pmol/L) among the total population of subjects with CM and a statistically significant mean difference was found using the paired *t* test (*p *=* *.003).

##### Age distribution of plasma β‐endorphin levels in the subjects with CM and controls

**Figure 1:**
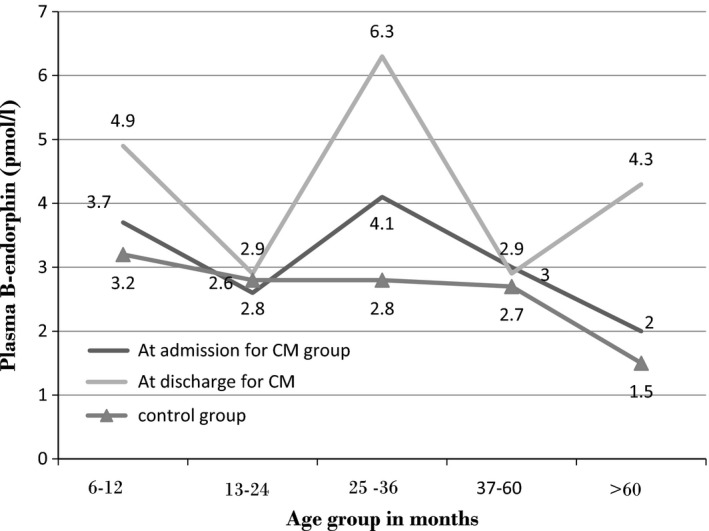
Age distribution of plasma β‐endorphin levels in the subjects with CM (at admission and discharge) and controls

Figure 1 shows that the plasma β‐endorphin level declined with increasing age among the control population. It was highest among the controls within age group 6–12 months but plateaus from age group 25–36 months and further declined for children above age 60 months. The plasma β‐endorphin was found to peak at age group 25–36 months for children with cerebral malaria both at admission and discharge.

#### 3.3.2 CSF β‐endorphin levels in subjects with CM at admission

The mean (±SD) CSF β‐endorphin level for subjects with CM at admission was 1.8 ± 0.9 pmol/L (range: 0.8–3.4 pmol/L). There was no statistically significant difference in CSF β‐endorphin level between the age groups as shown in Table 2 (F = 0.464, *p *=* *.761). The mean CSF β‐endorphin level in males (1.7 ± 0.9 pmol/L) showed no statistically significant difference from that of females (2.0 ± 0.9 pmol/L) (*p *=* *.405, *t *=* *−0.841).

**Table 2:** Mean CSF β‐endorphin levels in subjects with CM in relation to age groups

Variables	*N*	Mean ± SD	Range
CSF β‐endorphin			
6–12 months	4	1.7 ± 0.8	1.0–2.5
13–24 months	16	1.9 ± 1.0	0.9–3.4
25–36 months	10	1.9 ± 1.1	0.8–3.2
37–60 months	6	1.3 ± 0.6	1.0–2.6
>60 months	4	1.9 ± 0.9	1.0–2.9

One way ANOVA *F* = 0.464 *p* = .761.

##### Relationship between β‐endorphin levels and admission temperature

**Figure 2:**
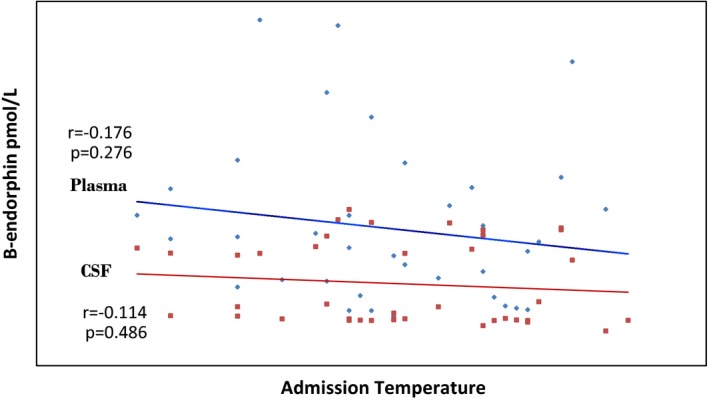
Relationship between plasma and CSF β‐endorphin levels and admission temperature. *r* = correlation coefficient, *R* ^2^ *= *coefficient of determination

Figure 2 shows that the plasma and CSF β‐endorphin levels were found to show a negative relationship with admission temperature but this was not statistically significant in both the cases (*p *=* *.276 and 0.486 for plasma and CSF β‐endorphin levels, respectively).

##### Relationship between β‐endorphin levels and fever clearance time

**Figure 3:**
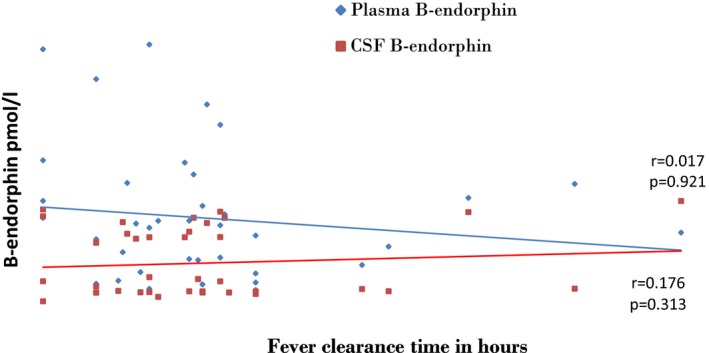
Relationship between β‐endorphin levels and fever clearance time

The plasma β‐endorphin was found to show no relationship with fever clearance time (*p*‐value (*r* = .017, *p*‐value .921) while CSF β‐endorphin was found to rise with increasing fever clearance time but this relationship was also not statistically significant (*r* = 0.176, *p*‐value 0.313) as shown in Figure 3.

#### 3.3.3 Outcome of cerebral malaria

##### Gender and outcome of cerebral malaria

Fifteen (60%) of the males in this study recovered fully as compared to five females (33.3%) but this was not statistically significant as shown in Table 3. Majority (60%) of the patients who died were females. Also, 46.7% of the females and 32% of males recovered with neurologic sequel. Majority of the 25 subjects with CM who recovered fully were males (75%) while only 25% were females. However, there was no significant association between gender and outcome of cerebral malaria (χ^2^ *= *2.95, df* = *2, *p = *.229).

**Table 3:** Relationship between gender and outcome of disease

Gender/Outcome	Full recovery	Recovery with sequelae	Death	Total
	*n* (%)	*n* (%)	*n* (%)	*n* (%)
Male	15 (60.0)	8 (32.0)	2 (8.0)	25 (62.5)
Female	5 (33.3)	7 (46.7)	3 (20.0)	15 (37.5)
Total	20 (50.0)	15 (37.5)	5 (12.5)	40 (100.0)

##### Beta endorphin levels and outcome of cerebral malaria

The patients that died had the highest CSF and plasma β‐endorphin level at admission compared with other forms of outcome as shown in Table 4. These were however not statistically significant (*p *=* *.614 and .194, respectively).

**Table 4:** Pattern of CSF and plasma β‐endorphin levels in relation to outcome

β‐endorphin levels	Outcome			*p* values^a^
	Full recovery	Recovered with sequel	Death	
	*n *= 20	*n *= 15	*n *= 5	
CSF level	1.7 ± 0.8	1.8 ± 1.0	2.2 ± 1.1	.614
Plasma level	3.3 ± 2.4	2.5 ± 1.4	4.3 ± 1.8	.194

*n *= frequency.

a One way ANOVA.

## 4 Discussion

This is the first study on CSF β‐endorphin levels in children with CM. The mean CSF β‐endorphin level obtained in our study was less than that found in the study by Nagamitsu (1993) on children with aseptic meningitis (10.95 ± 4.20 pmol/L). However, the small sample size of 16 subjects in the latter study relative to the sample size of 40 in our study makes comparison difficult. It is possible that different CNS infections may trigger release of CSF β‐endorphins to different degrees. There are only a few studies on CSF β‐endorphin level in children. Echenne et al. (1991; Nagamitsu et al., 1998) measured CSF β‐endorphin levels in 15 young girls with Rett syndrome and reported a mean value of 6.65 ± 3.45 pmol/L which was also higher than the value obtained in our study.

The mean CSF β‐endorphin levels among subjects with CM peaks between 25 and 36 months and is lowest between 37 and 60 months though there was no statistically significant difference in CSF β‐endorphin levels among the different age groups. These mean values are numerically different but not really far from each other on scale of measurement and the difference is not statistically significant. It may thus be due to chance. However, CSF levels of β‐endorphin have been observed in CNS infections to peak during the first year of life with a negative correlation with increasing age (Nagamitsu, 1993; Nagamitsu et al., 1998). For ethical reasons, CSF β‐endorphin was not determined in the controls in this study. Therefore, the CSF levels of β‐endorphin in the subjects with CM could not be compared with what obtains normally in the control population. For the same ethical reasons, CSF β‐endorphin levels were not determined following recovery from CM prior to discharge. The observation of high‐CSF β‐endorphin level in younger children has been explained on the basis of immaturity of the function of opioid receptors as well as immaturity of neurotransmitter networks resulting in increased availability and slower degradation of β endorphins (Nagamitsu, 1993). This may explain the declining plasma β endorphin levels with increasing age among the controls in this study.

The exact reason for the significant gender difference in plasma β‐endorphin levels among subjects with cerebral malaria but not in controls is unknown. The gender difference was only significant at discharge. We observed that poor outcome of CM was more common in females than males. It is therefore possible that the higher β‐endorphin levels observed among patients who died (majority of whom are females) compared to those who survived may be contributory to the gender difference in plasma β‐endorphin levels observed.

The higher plasma β‐endorphin level in the subjects with CM compared to controls is possibly due to overproduction of β‐endorphins triggered by cerebral hypoxia as well as stressors such as fever and acidosis associated with CM. Fever has been found to be a stressor that can lead to β‐endorphin production. Tsai, Lin, Wang, and Huang (2003) reported that pyrogens enhance β‐endorphin release in the hypothalamus and trigger fever. The converse is also probably true. For example, a study on unanesthetized rabbit, whose preoptic/anterior hypothalamus (POAH) were injected with β‐endorphin showed that β‐endorphin reduces sensitivity of POAH neurons to high ambient temperature and that this reduction leads to increase in peripheral vasoconstriction and inhibition of evaporative heat loss which leads to an elevation in body temperature (Gordon, Rezvani, & Heath, 1984) These findings suggest that β‐endorphins are thermogenic and may cause elevation in body temperature. CSF β‐endorphin levels were observed to rise with increasing fever clearance time (FCT) while a negative relationship was observed between admission temperature and β‐endorphin levels. This suggests that β‐endorphin level affects the duration and probably not the extent of fever.

In CM, acidosis is not unusual. It probably results from anaerobic glycolysis caused by microcirculatory obstruction leading to increased serum lactate levels. Acidosis as well as increased serum lactate has been reported to result in increased β‐endorphin production though this occurs mainly following exercise (Bender et al., 2007; Shephard, 2001). The same mechanism may be activated in patients with CM. It is noteworthy that low serum bicarbonate was observed in 42.5% of the subjects with CM. Plasma β endorphin level was also found to be significantly higher in the subjects with low serum bicarbonate. A more objective method of determining presence of acidosis through determination of base excess from arterial blood gas measurement may be helpful in determining contribution of acidosis to elevated β‐endorphin levels.

The elevated plasma β‐endorphin level seen in the subjects with CM was observed from samples taken both at admission and at discharge with a significantly higher mean at discharge than the level at admission. The mean plasma β‐endorphin of CM patients was significantly higher at discharge than at admission. The exact explanation for this observation is unknown. Since lumbar puncture was not done on CM patients at discharge for ethical reasons, no definite assertions can be made on the CSF β‐endorphin levels at discharge. It is known ,however, that CSF β‐endorphins have a longer half‐life than plasma β‐endorphins and that a retrograde transport of β‐endorphins from CSF to plasma via p‐glycoprotein may occur (Veening, Gerrits, & Barendregt, 2012). Perhaps, these may explain the raised plasma β‐endorphin levels at discharge. We think this may be a plausible reason since the subjects were not subjected to any stressful therapeutic procedure that can induce β‐endorphin production. We are not aware of any reports linking antimalarial treatment with β‐endorphin levels.

There is perhaps another explanation for the persistence of elevated plasma β‐endorphin levels at discharge compared to at admission. Most published works that suggest a short half‐life for plasma β‐endorphins were based on exercise‐induced stress and not following infections or inflammation (Shephard, 2001). Although there are qualitative similarities between the immune responses to exercise and sepsis, the magnitude of the changes induced by most forms of exercise remains much smaller than in a typical inflammatory response (Shephard, 2001). The main difference between the changes caused by sepsis and exercise appears to be of degree rather than type (Shephard, 2001).

In this study, plasma β‐endorphin levels were found to be higher than CSF levels which were similar to reports in earlier studies. Pituitary gland, peripheral tissues, and immunocytes of the immune system secrete β‐endorphin into the circulation which suggests that circulating β‐endorphin can hardly enter the brain ventricles (Machelska & Stein, 2000; Veening et al., 2012). Following its release from neurons β‐endorphin is partially destroyed at the synapses and in the interstitial fluid of the brain. These destructive sites may also explain why the β‐endorphin concentration in CSF was lower than in plasma. Hamel (1988) also found that CSF endorphin concentrations in man were significantly lower than in plasma. These results support the finding of a lower β‐endorphin concentration in CSF as compared with plasma in children with CM. In contrast, however, Jeffcoate et al. (1978), showed that β‐endorphin concentrations were higher in human CSF than in plasma.

Thus the findings from this study suggest an increased peripheral production of β‐endorphins in children with cerebral malaria during the course of the illness possibly from the effect of stressors like fever, hypoxia, and acidosis. The higher β‐endorphin level found in subjects who died compared to those who survived was not significant in this study. Thus we recommend that larger and prospective studies on CM be done with plasma β‐endorphin levels serially measured during the course of the illness and for some weeks after discharge to determine the pattern of rise of β‐endorphin levels during the course of the illness. Additionally, comparative studies of β‐endorphin levels in the key malaria syndromes and other CNS infections should be worthwhile. The subjects with other CNS infections will serve as nonhealthy controls.

## Conflict of Interest

The authors declare that there is no conflict of interest.
